# Sleep Apnea, Sleep Debt and Daytime Sleepiness Are Independently Associated with Road Accidents. A Cross-Sectional Study on Truck Drivers

**DOI:** 10.1371/journal.pone.0166262

**Published:** 2016-11-30

**Authors:** Sergio Garbarino, Paolo Durando, Ottavia Guglielmi, Guglielmo Dini, Francesca Bersi, Stefania Fornarino, Alessandra Toletone, Carlo Chiorri, Nicola Magnavita

**Affiliations:** 1 Department of Neuroscience, Rehabilitation, Ophthalmology, Genetics and Maternal/Child Sciences (DINOGMI), University of Genoa, Genoa, Italy; 2 Department of Health Sciences, Postgraduate School in Occupational Medicine, University of Genoa and Occupational Medicine Unit, IRCCS AOU San Martino IST, Genoa, Italy; 3 Department of Educational Sciences, University of Genoa, Genoa, Italy; 4 Institute of Public Health, Università Cattolica del Sacro Cuore, Rome, Italy; University of Rome Tor Vergata, ITALY

## Abstract

**Background:**

Recent research has found evidence of an association between motor vehicle accidents (MVAs) or near miss accidents (NMAs), and excessive daytime sleepiness (EDS) or its main medical cause, Obstructive Sleep Apnea (OSA). However, EDS can also be due to non-medical factors, such as sleep debt (SD), which is common among professional truck drivers. On the opposite side, rest breaks and naps are known to protect against accidents.

**Study Objectives:**

To investigate the association of OSA, SD, EDS, rest breaks and naps, with the occurrence of MVAs and NMAs in a large sample of truck drivers.

**Methods:**

949 male truck drivers took part in a cross-sectional medical examination and were asked to complete a questionnaire about sleep and waking habits, risk factors for OSA and EDS.

**Results:**

MVAs and NMAs were reported by 34.8% and 9.2% of participants, respectively. MVAs were significantly predicted by OSA (OR = 2.32 CI95% = 1.68–3.20), SD (OR = 1.45 CI95% = 1.29–1.63), EDS (OR = 1.73 CI95% = 1.15–2.61) and prevented by naps (OR = 0.59 CI95% = 0.44–0.79) or rest breaks (OR = 0.63 CI95% = 0.45–0.89). NMAs were significantly predicted by OSA (OR = 2.39 CI95% = 1.47–3.87) and SD (OR = 1.49 CI95% = 1.27–1.76) and prevented by naps (OR = 0.52 CI95% = 0.32–0.85) or rest breaks (OR = 0.49 CI95% = 0.29–0.82).

**Conclusions:**

When OSA, SD or EDS are present, the risk of MVAs or NMAs in truck drivers is severely increased. Taking a rest break or a nap appear to be protective against accidents.

## Introduction

In the last three decades or so, several studies demonstrated a clear relationship between excessive daytime sleepiness (EDS) and motor vehicle accidents (MVAs) [1–5]. EDS accounts for 20% of total MVAs, with lethality rates higher than those reported for MVAs associated with other risk-factors (11.4% *vs* 5.6%) [2]. Obstructive sleep apnea (OSA) is the main medical cause of EDS and is also associated with an increased risk of MVAs [6]. Unfortunately, professional drivers suffer from other risk factors for OSA. These include obesity, hypertension, dyslipidemia, smoking, drinking, and insufficient physical exercise [7,8], as well as gender (i.e., male) and increasing age [9,10]. Individuals with OSA show intermittent hypoxemia [11], reduced frontoparietal activation, failure in top-down prefrontal control and attentional networks [12]. These conditions tend to impair executive functions, alertness, sustained attention, and cognitive performance [13].

EDS in professional drivers can also be induced by other non-medical factors, such as sleep debt (SD), which, in turn, may be the result of organizational work factors (i.e., overtime, irregular schedules, night shifts), bad sleep habits (i.e., inadequate rest, extended wakefulness) and excessive physical and mental activity [14–19]. These factors are known to impair driving ability and induce subjective feelings of tiredness, slowed reaction times, lapses of attention to critical details and performance deterioration [20–23]. As a result, sleep- and EDS-related MVAs are more likely to occur among professional male drivers than in males in the general population [24,25].

Among road crashes, it has been reported that more than half of truck accidents cause fatal injuries and/or chronic disabilities and that the truck driver is found at fault in more than 80% of the cases [26]. Therefore, investigation of the risk and protective factors in truck drivers that maybe associated with MVAs and NMAs appears to be of crucial importance. Previous studies that addressed the above mentioned issue confirmed that EDS represent a specific risk factor for both MVAs and NMAs in this work category [27–29]. Nevertheless, to the best of our knowledge, no previous study simultaneously investigated OSA and EDS on one hand and SD on the other, in order to analyze the relative importance of these risk factors. The present study aims to fill this gap by also taking into account two behavioral countermeasures to sleepiness, such as naps [30] and rest breaks [31], that have already proved to be protective factors against accidents. In particular, the protective effect of these habits was investigated to confirm their specific contribution when associated with the above mentioned risk factors.

## Methods

### Recruitment of Participants

Between June 1, 2014 and May 31, 2015, a large modern trailer, parked in some of the major Italian trucking hubs (Turin, Novara, Verona, Bologna, Rome and Naples), was used as a mobile clinic to perform the survey. The vehicle comprised a fully-equipped clinic where medical examinations were carried out, a separate room for questionnaires administration and filling, and a relaxation area with a cafeteria and some internet terminals. A free and anonymous medical examination and standardized questionnaires focused on sleep-related disorders were respectively offered and administered to truck drivers by a well trained staff, composed of three/four medical doctors and two psychologists. Two nurses explained the purpose and the methods of the investigation to all the participants. Drivers also received a free vehicle check-up, consisting in an inspection of tires and lights, with free repairs when needed. This study was part of the "CNH Iveco Industrial Check-Stop Project", an international project for road safety, supported by the European Union Road Safety Action and the Italian Ministry of Transport.

All participants were informed about the aim of the study and signed an informed consent before participating in the survey. The study was approved by the Ethics Committee of the Liguria Region.

### Data Collection

Data were collected through medical examinations, semi-structured interviews and standardized questionnaires.

During the medical examination, basic demographic characteristics (i.e., gender and age), weight, height, and consumption of cigarettes and coffee were recorded. Medical history, clinical and physical evaluation were performed by professionals of the Centre of Sleep Medicine and the Postgraduate School in Occupational Medicine of the University of Genoa, Italy. Presence of known pathologies, body mass index (BMI), neck circumference [32], and Mallampati score (MS) [33,34]; were recorded and evaluated. The Mallampati score is a method originally used to predict the ease of endotracheal intubation. The score is assessed by asking the patient, in a sitting posture, to open his mouth and to protrude the tongue as much as possible (from class I in which it is possible to visualize soft palate, uvula, fauces, pillars visible, to class IV when the soft palate is not visible at all). Participants were interviewed about their sleeping habits, sleep hygiene, amount of hours of sleep, EDS, snoring, awakenings characterized by a sensation of suffocation, habit of taking rest breaks (15–30 minutes rest without sleep) and/or naps (sleeping for less than 30 minutes) during work. Smoking habit (number of cigarette packs a day) and coffee drinking habit (number of cups of coffee a day) were also recorded.

In addition, the study was documented with interviewer-administered questionnaires, and complete confidentiality was guaranteed during data collection and processing. The questionnaires used were the Epworth Sleepiness Scale [35,36] and the Berlin Questionnaire (BQ) [37]. The ESS is an 8-item questionnaire that provides a measure of EDS and average sleep propensity in daily life. ESS scores range from 0 (no daytime sleepiness) to 24 (the highest level of daytime sleepiness). The cut-off for EDS risk is 10 [38, 39]; scores between 10 and 15 indicated mild/moderate EDS and scores ≥16 severe EDS [35]. The ESS showed high internal consistency both in the original [38] and the Italian version [36].

The BQ is a 10-item questionnaire designed to assess three OSA risk categories: the presence and frequency of snoring behavior, wake time sleepiness or fatigue, a history of obesity (i.e., body mass index–BMI≥ 30 kg/m^2^) and/or hypertension. It has been translated into Italian and validated [40–42], A driver was considered as being at high risk for having OSA if he scored "positive" in two or more sections of the BQ, and had one or more of the following characteristics and conditions: BMI>30kg/m^2^; neck circumference >42 cm; Mallampati score > 2. Subjects classified in the high-risk group have an Apnoea–Hypopnoea Index (AHI) > 5/h with a sensitivity of 0.86, a specificity of 0.77 and a positive predictive value of 0.89 [37]. Clinical diagnosis of suspect OSA [43] was based upon craniofacial and upper airway morphological characteristics, BMI, neck circumference, and BQ results.

Sleep Debt (SD) was defined as the difference between the amount of sleep you should be getting and the amount you actually get [44]. We calculated SD making 2 answers: “On average, how many hours of sleep do you get in a 24-hour period? Think about the time you actually spend sleeping or napping”; and: “On average, how many hours of sleep do you think you should get in a 24-hour period?”. We computed SD by subtracting the slept hours reported from the desired hours of sleep, and used it as a continuous variable. All the above reported variables were investigated as predictors in the risk analyses.

The occurrence of MVAs in the previous three years, and of NMAs in the previous six months, was surveyed using the following yes/no questions: "Did you have a motor vehicle accident at work during the last three years?", "Did you have a near-miss driving accident during the last six months?".

### Statistics

Descriptive statistics were computed as mean ± standard deviation for continuous variables and as frequencies and percentages for categorical variables.

Logistic regression analysis was firstly used to investigate the univariate association between the predictors and the response variables. Odds ratio (OR) and their 95% confidence interval (95% CI) were computed.

Multiple hierarchical logistic regression analyses, with MVAs and NMAs as response variables, were then performed to investigate the independent role of each predictor. In Model I only background variables (age, cigarettes and coffee consumption) were entered as predictors. In Model II the clinical variable, i.e., suspect OSA, was entered. In Model III we entered sleep debt and in Model IV we entered its possible consequence, EDS. In Model V we entered one of the countermeasures against sleepiness (i.e., rest breaks), while in Model VI the other countermeasure, (i.e., naps) was considered. These latter two models allowed us to investigate the protective effect of these behaviors against MVAs and NMAs after risk factors had been accounted for.

Statistical analyses were performed with IBM/SPSS 20.0.

## Results

A total of 1,540 truck drivers were contacted and informed of the study; 949 (61.6%) performed the clinical examination and completed the questionnaires according to the study methods and were properly enrolled in the survey. All subjects recruited were men, with a mean age of 44.30 (*SD* = 10.15). The descriptive statistic of the predictors and response variables investigated is outlined in Table 1. Nearly a quarter (230, 24.3%) of the participants had a SD of two or more hours. MVAs occurred in the previous three years were reported by more than one third of the participants. NMAs occurred in the previous six months were reported by almost 10% of the study population.

**Table 1:** Demographic and clinical characteristics of the participants (*n* = 949).

Variable	Statistic
Age (M±SD)	44.30±10.15
BMI	28.10 ± 4.72
Neck circumference	40.89 ±4.35
Mallampati score	
I	288 (30.3%)
II	349 (36.8%)
III	211 (22.2%)
IV	101 (10.6%)
Coffee consumption (cups, M±SD)	3.24±2.05
Cigarette consumption (packs, M±SD)	0.41±0.60
Suspect OSA [N (%)]	245 (25.8%)
Sleep debt (hours, M±SD)	0.91±1.22
Daily sleep debt of two or more hours	230 (24.3%)
EDS [N (%)]	127 (13.4%)
Rest breaks [N (%)]	748 (78.8%)
Naps [N (%)]	422 (44.5%)
MVAs [N (%)]	330 (34.8%)
NMAs [N (%)]	87 (9.2%)

M: Mean; SD: Standard deviation; N: Number; (%): Percentage; Suspect OSA: Obstructive sleep apnea, as evaluated with the Berlin Questionnaire and medical examination; Sleep debt: reported hours of sleep—desired hours of sleep; EDS: excessive daytime sleepiness, as evaluated by the Epworth Sleepiness Scale; MVAs: motor vehicle accidents; NMAs: near miss accidents

**Fig 1:**
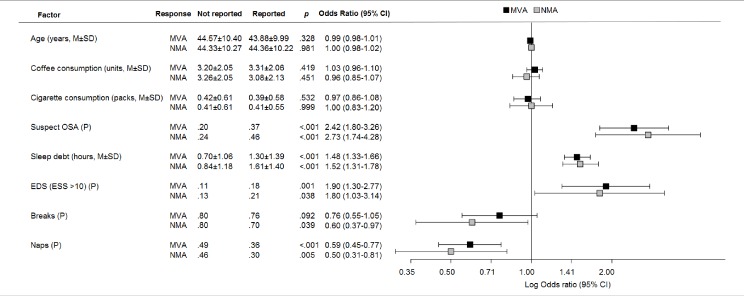
Bivariate associations of motor vehicle accidents (MVAs) and near miss accidents(NMAs) with the risk and protective factors investigated in this study. Note *M*: mean; *SD*: standard deviation; *P*: proportion. Suspect OSA: Obstructive sleep apnea, as evaluated with the Berlin Questionnaire and medical examination; Sleep debt: reported hours of sleep—desired hours of sleep; EDS: excessive daytime sleepiness, as evaluated by the Epworth Sleepiness Scale; MVAs: motor vehicle accidents; NMAs: near miss accidents.

Background variables were not associated with either MVAs or NMAs (Fig 1): suspect OSA, sleep debt and EDS emerged as significant risk factors for both the response variables. While results showed that taking naps was a significant protective factor against both MVAs and NMAs, the same result was obtained for a rest break only against NMAs.

**Fig 2:**
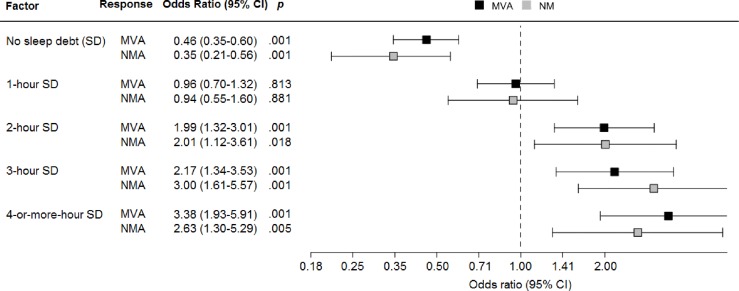
Relationship between sleep debt and motor vehicle accidents (MVAs) or near miss accidents (NMAs) in truck drivers.

**Fig 3:**
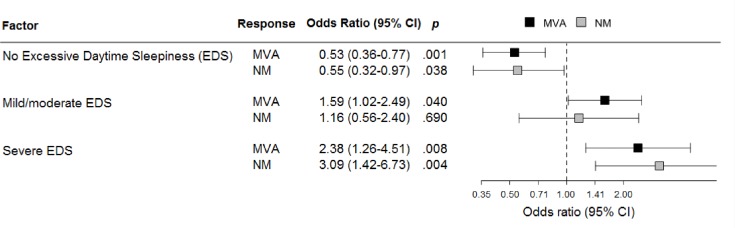
Relationship between excessive daytime sleepiness (EDS) and motor vehicle accidents (MVAs) or near miss accidents (NMAs) in truck drivers.

Increasing the SD and the severity of EDS, the risk of MVAs and NMAs linearly increased (Figs 2 and 3).

As might have been expected from the results outlined in Fig 1, background variables were not significant predictors of MVAs (Table 2) in the hierarchical multiple logistic regressions analysis.

**Table 2:** Hierarchic logistic regression analysis. Relationship between predictor variables investigated and motor vehicle accidents (MVAs).

Variable	Model I	Model II	Model III	Model IV	Model V	Model VI
	OR (95%CI)	OR (95%CI)	OR (95%CI)	OR (95%CI)	OR (95%CI)	OR (95%CI)
**Age**(years)	0.99 (0.98–1.01)	0.99 (0.97–1.01)	0.99 (0.97–1.01)	0.99 (0.97–1.01)	0.99 (0.97–1.01)	0.99 (0.97–1.01)
**Coffee drinking**(cups/day)	1.05 (0.98–1.13	1.04 (0.97–1.12)	1.02 (0.95–1.10)	1.02 (0.95–1.10)	1.02 (0.95–1.11)	1.02 (0.95–1.11)
**Smoking**(packs/day)	0.95 (0.84–1.08)	0.94 (0.83–1.06)	0.97 (0.86–1.11)	0.97 (0.86–1.11)	0.98 (0.86–1.12)	0.98 (0.86–1.11)
**SuspectOSA**		2.61 (1.91–3.56)***	2.47 (1.80–3.39)***	2.30 (1.67–3.18)***	2.37 (1.71–3.27)***	2.32 (1.68–3.20)***
**Sleepdebt**(hours)			1.44 (1.28–1.62)***	1.45 (1.30–1.62)***	1.47 (1.31–1.65)***	1.45 (1.29–1.63)***
**EDS** (ESS >10)				1.68 (1.12–2.52)*	1.68 (1.12–2.52)*	1.73 (1.15–2.61)**
**Rest breaks**					0.63 (0.45–0.89)**	
**Naps**						0.59 (0.44–0.79)***
**R**^**2**^	0.005	0.06	0.11	0.12	0.13	0.14

Significance

*: < 0.05

** <0.01

*** = p<0.001.

Model 1 = Demographic and lifestyle variables; Model II: including pathology; Model III: including sleep debt; Model IV: including EDS; Models V-VI: including remedies.

Note OR: odds ratio; 95%CI: confidence interval; R^2^: determination coefficient. Suspect OSA: Obstructive sleep apnea, as evaluated with the Berlin Questionnaire and medical examination; Sleep debt: reported hours of sleep—desired hours of sleep; EDS: excessive daytime sleepiness, as evaluated by the Epworth Sleepiness Scale; MVAs: motor vehicle accidents; NMAs: near miss accidents

Risk for OSA, perceived SD and EDS were all significant risk factors. Having a rest break was associated with a slight, but significant reduction of the risk of MVAs in Model V, while taking a nap was a significant protective factor in model VI, where rest breaks were not considered.

A very similar pattern of results was observed for NMAs. Background variables were not significant predictors, while risk for OSA, perceived SD and risk for EDS were all significant risk factors, as well as rest breaks and naps were protective factors, with a stronger effect for the latter (Table 3).

**Table 3:** Hierarchic logistic regression analysis. Relationship between predictor variables investigated and near miss accidents (NMAs).

Variable	Model I	Model II	Model III	Model IV	Model V	Model VI
	OR (95%CI)	OR (95%CI)	OR (95%CI)	OR (95%CI)	OR (95%CI)	OR (95%CI)
**Age**(years)	1.01 (0.98–1.02)	0.99 (0.97–1.02)	0.99 (0.97–1.02)	0.99 (0.97–1.02)	1.00 (0.97–1.02)	0.99 (0.97–1.02)
**Coffee drinking**(cups/day)	0.95 (0.84–1.10)	0.94 (0.83–1.06)	0.91 (0.80–1.04)	0.91 (0.80–1.04)	0.92 (0.81–1.05)	0.92 (0.81–1.04)
**Smoking**(packs/day)	1.04 (0.85–1.26)	1.02 (0.84–1.25)	1.90 (0.89–1.33)	1.09 (0.89–1.33)	1.10 (0.89–1.35)	1.09 (0.89–1.34)
**SuspectOSA**		2.74 (1.73–4.34)***	2.51 (1.57–4.02)***	2.37 (1.46–3.82)***	2.48 (1.53–4.03)***	2.39 (1.47–3.87)***
**Sleepdebt**(hours)			1.49 (1.27–1.75)***	1.50 (1.27–1.76)***	1.53 (1.30–1.81)***	1.49 (1.27–1.76)***
**EDS** (ESS >10)				1.52 (0.85–2.74)	1.50 (0.83–2.72)	1.54 (0.85–2.78)
**Rest breaks**					0.49 (0.29–0.82)**	
**Naps**						0.52 (0.32–0.85)**
**R**^**2**^	0.001	0.04	0.09	0.10	0.11	0.11

Significance

*: < 0.05

** <0.01

*** = p<0.001

Model 1 = Demographic and lifestyle variables; Model II: including pathology; Model III: including sleep debt; Model IV: including EDS; Models V-VI: including remedies.

Note OR: odds ratio; 95%CI: confidence interval; R2: determination coefficient. Suspect OSA: Obstructive sleep apnea, as evaluated with the Berlin Questionnaire and medical examination; Sleep debt: reported hours of sleep—desired hours of sleep; EDS: excessive daytime sleepiness, as evaluated by the Epworth Sleepiness Scale; MVAs: motor vehicle accidents; NMAs: near miss accidents

## Discussion

This study aimed at investigating some risk and protective factors for MVAs and NMAs, and their interaction for the occurrence of such events. The results indicated that risk for OSA, SD and EDS have a significant impact on truck drivers safety considering both the outcomes investigated. Furthermore, we also demonstrated that the routine use of sleepiness countermeasures, such as taking a nap or a rest break during driving, play a protective effect in truck drivers at risk.

One out of four truck drivers enrolled in the study resulted at risk for OSA: although this finding should be interpreted with caution, on account of the self-selection of participants, this proportion is consistent with the results of recent studies reporting a prevalence of suspect OSA in professional drivers ranging between 15% and 56% [9,45]. Thus, OSA is confirmed to be a critical safety concern in professional drivers. We provided further evidence that drivers at high risk for OSA are also at increased risk for both MVAs and NMAs. A recent systematic review and meta-analysis of the literature, investigating the relationship between OSA and working accidents, showed that OSA patients have an almost double risk of injuries, and that professional drivers have a higher OR increase than other occupational categories for this outcome [46].

We also found that sleep deprivation had a significant association with driving accidents after the effect of OSA has been ruled out, with almost a quarter of the participants reporting this condition for two or more hours. Few studies analyzed the association between MVAs, NMAs and SD in professional drivers. Johnson et al. [29] observed that known risk factors for poor sleep or EDS were significantly associated with self-reported MVAs and/or NMAs in transportation drivers (i.e., driving drowsy OR = 4.1; CI, 2.5 to 6.7). These authors also observed that adequate sleep duration (more than 7 hours) was associated with a 40% decrease in the risk of a self-reported accident or near miss accident (OR = 0.6; CI, 0.4 to 0.9). Carter et al. [24], using a measure of SD similar to that used in our study, found that self-perceived SD was directly related to accident occurrence in males, both in the general population and in professional drivers. Our results are also consistent with those of other studies on car drivers that demonstrated an association between the amount of hours of sleep per night and MVA [47] or NMA rates [48]. A recent population-based case-control study reported that drivers with 6 hours or less of nocturnal sleep during the previous 3 months were at significantly increased risk of MVAs (> 69%) when compared to those who had slept for more than 6 hours [49]. In another study, short habitual sleep time (≤ 5 hours/night) was associated with a 2.7-fold increased risk of motor vehicle accidents [6].

Our finding of a 13% rate of EDS is consistent with previous studies showing that daytime sleepiness in professional drivers ranges between 13 and 20% [27,29,50,51]. Our study also showed that EDS was a significant predictor of MVAs, after the effect of OSA and SD had been ruled out. This finding suggests that a higher risk of MVAs cannot be adequately explained by OSA and SD, and that EDS is a further, independent risk factor. Despite the role of sleepiness in road traffic accidents has already been pointed out in previous studies [50], to the best of our knowledge, no Author attempted to separate the relative effects of EDS from that of OSA and SD. Even if the most prevalent cause of sleepiness-related accidents is the behaviourally induced sleep insufficiency syndrome or irregular sleep-wake rhythm in otherwise healthy subjects [50], and OSA or other medical conditions are other well-known causes of EDS, our study suggests that other possible causes of EDS, such as the use of medication, inappropriate eating and/or drinking, other sleep disorders, medical and psychiatric comorbidities may be of concern for the occurrence of this condition and should be taken into account.

Among commonly proposed sleepiness countermeasures, we observed that drinking a coffee had no significant effect, while the effect of taking a nap proved to be significant after the effect of risk factors had been ruled out. This result suggests that the rest break is more likely to be beneficial if the driver sleeps for a while during the stop. On the other hand, taking a rest break was a significant countermeasure, even if less efficient than taking a nap. This observation has much comforted us about the effectiveness of our health promotion activity. In fact, even the truck drivers who just done the free vehicle checking, without completing the proposed questionnaires and medical examination, have had a benefit from the stop in the service area if they were tired or had sleep problems.

It should be noted that our prediction models of MVAs and NMAs cannot be considered to be exhaustive, as those outcomes are multi-causal events in which sleepiness and other sleep-related variables are only some of the factors. The inclusion of other medical and non-medical causes of EDS, which we have not considered in this study, could probably provide better prediction models. This study, however, demonstrated that medical and non-medical risk factors of EDS may play a significant role in MVAs, and that their importance is undoubtedly higher than estimated in official statistics, which generally attribute only 1–2% of accidents to sleep-related factors [50,51].

One of the main implications of the results of this study is the role played by sleep-related factors in the occurrence of MVAs. Unlike other risk factors such as alcohol and drugs, being sleepy is not against the law, and it is not investigated by traffic corps at routine roadblocks. Hence, the problem was overlooked for years until the European Commission issued a Directive requesting mandatory testing for OSA and EDS in all European countries before a driver's license is granted or renewed, in 2015 [52].

Some limitations of this study need to be pointed out. Since it was a cross-sectional study, the design itself prevented any conclusive causal inference. Hence, the chain of events assumed in the hierarchical multiple logistic regression models is only presumptive, albeit based upon previous studies and biological plausibility.

Furthermore, the self-selection of the participants limited the generalizability of the results to the actual population of truck drivers. Consequently, the prevalence of the problems observed cannot be considered as a consistent estimate of that in the entire working category. However, we have no reason to believe that self-selection may have influenced the observed associations of risk and protective factors with the outcomes investigated. The retrospective character of the study, with a 3-year recall period for MVAs and 6-month recall period for NMAs, may have introduced a recall bias that may be especially relevant for the latter, poorly documented, events. However, MVAs and NMAs are relatively low-frequency events, hence a shorter time frame would not have provided enough variability in the outcome variables. On the other hand, given the importance of these events, their recall is likely to have been accurately reported.

Besides the large number of the participants, the main strength of this study was that drivers knew that the data they provided would not have any consequences for their job, since they would have not been reported to employers, supervisors or authorities. This definitely encouraged honesty in reporting and enhanced the reliability of the results collected. Moreover, the assessment of suspect OSA and EDS was performed with standardized methods. To the best of our knowledge, this survey is the largest performed in Italy, and one of the largest worldwide, among the studies on sleep factors and driving accidents conducted directly in the workplace. A recent review, that underlined the scarce number of well-conducted studies on this issue, observed that the main reason seemed to be that OSA was not considered an occupational disease and it is consequently neglected by the occupational health services and transport companies [46].

We claim that screening for OSA, EDS, and SD should be a primary prevention task of the occupational physician when evaluating health of professional drivers. The early identification of individuals at risk for these conditions through systematic screening would improve workers’ health and well-being, and might reduce the number of driving accidents, with clear benefits for the safety of workers and third parties, as well as economic advantages for transport companies and society as a whole. Educational programs focused on sleep hygiene and on preventive measures against falling asleep at the wheel could also be useful for improving not only individual safety but also public health.
